# Experimental Cancer Medicine Centre (ECMC) network proposal for a consensus gene panel for pan-cancer sequencing: a Delphi methodology

**DOI:** 10.1038/s41416-025-03252-6

**Published:** 2025-11-08

**Authors:** Richard Phillips, Bristi Basu, Zohra Butt, Mounia Beloueche, Natalie Cook, Simon J. Crabb, Alastair Greystoke, Darren Hargrave, Robert Jones, Muhammad Yasin Kureeman, Juanita Lopez, Claire McKeeve, Magdalena Meissner, Brent O’Carrigan, Eileen E. Parkes, Milind Ronghe, Patricia Roxburgh, Debashis Sarker, Kienan I. Savage, Paul H. S. Shaw, Stefan Symeonides, Deborah A. Tweddle, Aditi Vedi, Harriet S. Walter, Joanna Zabkiewicz, Gary W. Middleton, Andrew D. Beggs

**Affiliations:** 1 Department of Cancer & Genomic Sciences, College of Medicine and Health, University of Birmingham, Birmingham, UK; 2 University of Cambridge and Cambridge University Hospitals NHS Foundation Trust, Cambridge, UK; 3 Pierre Fabre Ltd, Green Park, Reading UK; 4 Boehringer Ingelheim, Ingelheim am Rhein, Germany; 5 Experimental Cancer Medicine Team, Christie Hospital NHS Foundation Trust and University of Manchester, Manchester, UK; 6 Southampton ECMC, University of Southampton, Southampton, UK; 7 Institute of Clinical and Translational Medicine, Newcastle University, Newcastle upon Tyne, UK; 8 Paediatric Oncology, University College London Institute of Child Health, London, UK; 9 Cardiff University and Velindre University NHS Trust, Velindre School of Medicine, Museum Avenue, Cardiff, UK; 10 Drug Development Unit, Royal Marsden NHS Foundation Trust and the Institute of Cancer Research, Oak Cancer Centre, Sutton, UK; 11 West of Scotland Centre for Genomic Medicine, NHS Greater Glasgow and Clyde, Glasgow, UK; 12 Department of Oncology, University of Oxford, Oxford, UK; 13 Paediatric Oncology Department, Royal Hospital for Children, Glasgow, UK; 14 School of Cancer Sciences, Wolfson Wohl Cancer Research Centre, University of Glasgow, Glasgow, UK; 15 School of Cancer and Pharmaceutical Sciences, King’s College London, London, UK; 16 Patrick G Johnston Centre for Cancer Research, Queen’s University Belfast, Belfast, UK; 17 Department of Clinical Oncology, Velindre University NHS Trust, Cardiff, UK; 18 Edinburgh Experimental Cancer Medicine Centre, Institute of Genetics and Cancer, University of Edinburgh, Edinburgh, UK; 19 University Hospitals of Leicester NHS Trust, Leicester, UK; 20 Department of Haematology, School of Medicine, Cardiff University, Heath Hospital, Cardiff, UK

**Keywords:** Cancer genomics, Genetics research, Cancer genomics, Cancer genomics, Cancer genetics

## Abstract

**Background:**

The Experimental Cancer Medicine Centre (ECMC) Network supports UK-wide access to experimental cancer therapies, many of which require specific genomic alterations. This study aimed to develop expert consensus on essential genes for a pan-cancer sequencing panel, involving subject matter experts (SMEs) from the ECMC Network and the pharmaceutical industry.

**Methods:**

A pilot with 8 SMEs graded 526 genes, refining the list to 210. A three-round Delphi process was then used, with SMEs iteratively evaluating each gene. In the final round, SMEs also assessed the inclusion of tumour mutational burden (TMB), microsatellite instability (MSI), and mutation types (structural variations, copy number variations, and/or fusions).

**Results:**

Consensus was reached on a final panel of 99 genes applicable across multiple cancers. High agreement was also achieved for including TMB, MSI, and screening for structural variations, copy number variants, and fusions. The panel is intended for both adult and paediatric tumour types.

**Conclusions:**

This consensus-based gene panel offers a standardised approach to pan-cancer genomic screening. It supports harmonised diagnostics and could improve patient access to personalised therapies and research trials across clinical and NHS settings.

## Introduction

In the UK, cancer genetic testing is primarily conducted through targeted panels that focus on specific cancer types or known genetic mutations associated with particular cancers, such as *BRCA1* and *BRCA2* mutations for breast and ovarian cancers [1].

In England, these tests are generally run within the framework of the NHS Genomic Medicine Service. This is divided into seven regional genomic laboratory hubs (GLHs), which are each responsible for coordinating genomic services for a particular part of the country [2]. This approach ensures a consistent, high-quality system across England, allowing the NHS to leverage genetic information to guide treatment decisions and facilitate early diagnosis. However, no standardised, pan-cancer gene panel has been widely implemented across these GLHs, and the number of genes screened varies depending on the cancer type and the laboratory’s chosen protocols. In the devolved nations, high quality genomic testing is provided by a variety of delivery models and the lack of a consistent pan-cancer panel that is seen in England persists. Provision of a pan-cancer panel through NHS labs, rather than relying on proprietary panels, such as Illumina 500 or Foundation One, would ensure greater accessibility and affordability for the UK population, making it possible to provide equitable care across public health settings. However, it requires identification of a unified panel in order to prevent inter-lab variability. Currently, there is no standardised panel with expert agreement over which genes should be incorporated.

The importance of defining a pan-cancer panel is underscored by the success of targeted therapy trials, which rely on identifying patients with specific genetic alterations. For example, the Lung MATRIX trial, one of the largest precision medicine trials in non-small cell lung cancer, has successfully matched patients to targeted therapies based on genomic profiling [3]. Similarly, the detection of *NTRK* fusions has enabled tumour-agnostic treatment approaches using larotrectinib, a TRK inhibitor with high response rates across multiple cancer types [4]. These examples illustrate how broad genetic screening can open the door to transformative treatment options, provided the right mutations are identified reliably. A well-designed pan-cancer panel could streamline this process by ensuring that clinically relevant and actionable mutations are not missed during testing.

The Molecular Diagnostics Laboratory at The Royal Marsden Hospital (RMH) in the UK have derived a comprehensive next-generation sequencing (NGS) assay of 233 protein-coding genes, termed the RMH200 Solid Tumour DNA NHS Panel version 2. However, this may not include all the genes relevant to trial research and may be too large to scale up to a nation-wide setting. While whole exome or genome sequencing might appear attractive for pan-cancer screening, in practice, these approaches remain limited by high costs, data complexity, and insufficient read depth to confidently detect low-frequency variants, especially in impure or heterogeneous tumour samples. These limitations underscore the need for a more focused, high-confidence panel approach that can deliver clinically useful results at scale within the constraints of NHS pathology workflows and budgets.

Efforts to incorporate genomic sequencing into cancer care and research have been bolstered by the 100,000 genomes project, and the question is now turning to how genomic sequencing will be adopted and used within oncological research [5]. Adopting a pan-cancer sequencing approach would bring significant benefits to cancer research and patient care.

A universal panel that screens for a broad set of genes associated with various cancers could streamline genetic testing, enabling quicker, more cost-effective analysis and make it easier to compare genetic data across cancer types. Additionally, this approach could allow for more personalised treatment options, and assist with directing potential participants to suitable research trials.

## Methods

### Pilot

8 subject matter experts (SMEs) were asked to grade 526 genes as “essential”, “desirable” or “not essential/desirable” for the screening tool. The 526 genes were collated from an overlap of all genes included in common commercially available arrays. 164 genes were recorded as “essential” by one or more experts, with 126 genes graded as “desirable”. 206 genes received at least one of these gradings. An additional 4 genes were proposed as free-text option to be part of the panel. This produced a list of 210 genes, which were used in the round 1 questionnaire of the Delphi process.

### Subject matter expert inclusion criteria

SMEs were approached through their involvement within the UK Experimental Cancer Medicine Centre (ECMC) Network through targeted emailing to each institutional representative. SMEs comprised cancer specialists across a range of specialities, including adult and paediatric solid tumour types. A snowballing method was then employed by asking those representatives to share the survey with any other members within their local network that they would deem appropriate to be included. Genomics pharmaceutical companies were also contacted.

### Delphi process

63 SMEs were invited to participate in a Delphi process to iteratively vote on the inclusion of each of 210 genes for a final pan-cancer screening panel. A Delphi process was selected because this structured technique enabled an objective discussion between SMEs and also ensured unbiased, balanced communication from all parties. Its ability to resolve disagreement through consensus agreement led to the formulation of a recommendation on which genes should be included.

### Data collection from main Delphi process

210 genes were organised via cellular functions via Gene Ontology (GO) consortium and formatted into a matrix survey using the software, Redcap. Any gene which was voted for inclusion by >60% respondents in a round was accepted into the subsequent round of Delphi. Closing criteria for each round was time-based (<2 months per round) with an aim of >50% respondent rate from those contacted. This project ran for 3 rounds of the Delphi and reached consensus at round 2. Once consensus was reached, a final round was conducted to ask SMEs to vote on whether to include tumour mutational burden (TMB) and microsatellite instability (MSI) within the panel, as well as whether to screen for structural variations (SVs), copy number variations (CNVs), and/or fusions for each gene.

## Results

### Cohort characteristics

The round 1 survey was responded to by 37 SMEs, including ECMC Network leads and pharmaceutical genomics industry representatives (59% of the SMEs who were originally contacted). Round 2 had 28 respondents (75.7% of respondents from Round 1). Round 3 had 25 respondents (89.3% of respondents from Round 2).

Of the final respondents, 82% had expertise in solid tumours in adults, 12% in solid tumours in paediatrics and 16% in haematological malignancies.

### Consensus

The number of genes that reached a 60% consensus in round 1 was 106 of 210 genes (50.5%). As such, 106 genes progressed into round 2, where 99 of these genes reached the 60% consensus threshold (93.4%). As such, these 99 genes were selected for the panel (Table 1).

**Table 1:** Final list of 99 recommended genes to be included in an ECMC-approved pan-cancer panel.

*AKT1*	*CDH1*	*FGFR4*	*MTOR*	*RAD51*
*AKT2*	*CDK4*	*FLT1*	*MUTYH*	*RAD51B*
*AKT3*	*CDK6*	*FLT3*	*MYC*	*RAD51C*
*ALK*	*CDK12*	*HRAS*	*MYCN*	*RAD51D*
*APC*	*CDKN2A*	*HLAA*	*NF1*	*RB1*
*AR*	*CDKN2B*	*IDH1*	*NF2*	*RET*
*ARID1A*	*CHEK1*	*IDH2*	*NRAS*	*ROS1*
*ARID1B*	*CHEK2*	*KRAS*	*NRG1*	*SETD2*
*ATM*	*CTNNB1*	*KIT*	*NTRK1*	*SMAD4*
*ATR*	*EGFR*	*JAK1*	*NTRK2*	*SMARCA4*
*ATRX*	*ERBB2*	*JAK2*	*NTRK3*	*SMARCB1*
*BAP1*	*ERBB3*	*JAK3*	*PALB2*	*STK11*
*BCL2*	*ERBB4*	*MDM2*	*PARP1*	*TERT*
*BRAF*	*ESR1*	*MDM4*	*PDGFRA*	*TET2*
*BRCA1*	*EZH2*	*MEN1*	*PDGFRB*	*TP53*
*BRCA2*	*FANCA*	*MET*	*PIK3CA*	*TSC1*
*BRIP1*	*FANCC*	*MLH1*	*PMS1*	*TSC2*
*CCND1*	*FGFR1*	*MSH2*	*PMS2*	*VEFGA*
*CCND2*	*FGFR2*	*MSH3*	*POLE*	*VHL*
*CCNE1*	*FGFR3*	*MSH6*	*PTEN*	

**Fig. 1:**
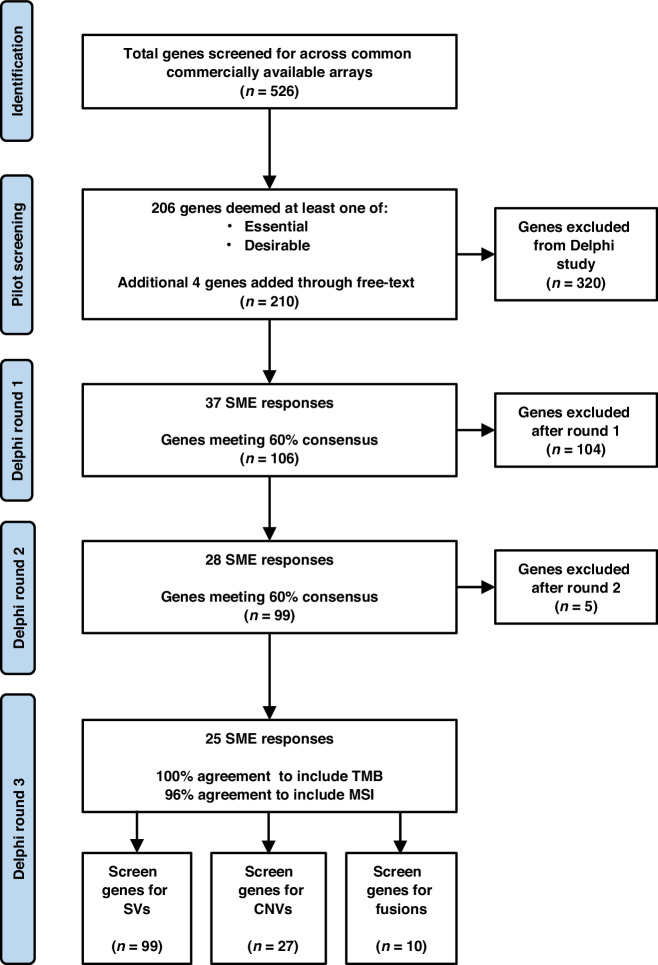
Consort flowchart demonstrating the pilot and Delphi study process. SME (subject matter expert), SVs (structural variations), CNVs (copy number variations)

In round 3, SMEs voted on whether to include tumour mutational burden (TMB) and microsatellite instability (MSI) within the panel, which were selected for with 100% and 96% consensus, respectively. Additionally, for each of the 99 genes, respondents voted on whether to screen for SVs, CNVs, and/or fusions. All 99 genes reached a consensus threshold for assessment of SVs; 27 genes were recommended to be screened for CNVs (Table 2) and 11 genes for fusions (Table 3). The process of the Delphi and exclusions at each round are demonstrated in Fig. 1.

**Table 2:** 27 genes from the list of 99 which should be screened for copy number variations as part of the ECMC-approved pan-cancer panel.

*AKT1*	*CDK4*	*ERBB2*	*MET*
*AKT2*	*CDK6*	*ERBB4*	*MYC*
*ALK*	*CDKN2A*	*FGFR1*	*MYCN*
*AR*	*CDKN2B*	*FGFR3*	*PTEN*
*CCND1*	*CTNNB1*	*FGFR4*	*RB1*
*CCND2*	*EGFR*	*KIT*	*TERT*
*CCNE1*	*ERBB1*	*KRAS*

**Table 3:** 11 genes from the list of 99 which should be screened for fusions as part of the ECMC-approved pan-cancer panel.

*ALK*	*FGFR2*	*NTRK1*	*RET*
*BRAF*	*FGFR3*	*NTRK2*	*ROS*
*FGFR1*	*MDM2*	*NTRK3*	

## Discussion

The development of a consensus-based pan-cancer large gene panel for UK cancer genomics research represents a key advancement toward standardising genetic testing across cancer types, which may ultimately streamline treatment pathways and access to research trials. This study’s three-round Delphi process enabled iterative evaluation by subject matter experts (SMEs), resulting in a final list of 99 genes deemed essential for broad-spectrum genomic screening of tumours. Consensus was also achieved on the inclusion of significant genomic markers, such as tumour mutational burden (TMB) and microsatellite instability (MSI), which hold established relevance in multiple cancer types. Through collaboration across the UK’s ECMC Network and involvement of SMEs from academia and industry, this study addresses a critical need to unify and streamline gene panel protocols, providing a foundation for comprehensive cancer genomic screening in the NHS.

**Fig. 2:**
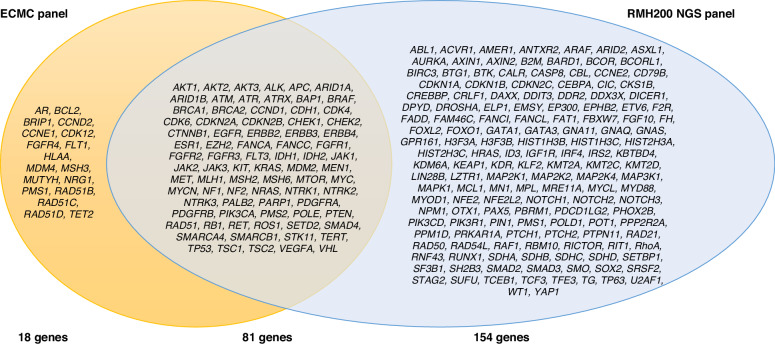
Comparison of the genes included within the RMH200 NGS panel and the proposed ECMC-approved pan-cancer panel

The results underscore the importance of a pan-cancer approach, aligning with emerging research advocating for broad-spectrum genetic screening as a means to capture common and unique mutations across various cancers [6]. Large pan-cancer panels have the potential to inform personalised medicine by identifying actionable mutations and biomarkers that transcend single-cancer paradigms, offering patients tailored treatment opportunities and more accurate prognostic assessments. Unlike limited, tumour-specific panels, a large pan-cancer panel approach may facilitate a more unique diagnosis, equitable patient access, and streamlined entry into clinical trials for targeted therapies. Integrating such a panel within the NHS could standardise patient access and provide reliable infrastructure without reliance on proprietary external panels, such as the Illumina 500 or Foundation One panels, ensuring consistency and affordability across UK cancer care settings. A comparison between the ECMC-derived panel and the existing RMH200 NGS panel shows areas of both overlap and divergence. While 81 genes are shared between the two panels, the ECMC panel also includes an additional 18 genes deemed critical for research relevance and trial stratification, reflecting broader pan-cancer applicability. Conversely, 154 genes included in the RMH panel were deprioritised by consensus in this Delphi process due to either low perceived utility or tumour-specific limitations. This comparison (Fig. 2) underscores the ECMC panel’s focus on a streamlined, high-utility gene set with strong translational relevance, and reinforces its potential for scalable deployment within the NHS. The clinical application of each gene is included in Supplementary Table 1.

Despite the robust outcomes achieved, the Delphi methodology presents limitations inherent to expert-driven consensus models. The Delphi approach relies on repeated rounds of expert surveys, which may lead to participant fatigue, especially given the panel’s size and complexity. In this study, survey response rates decreased by each round, from 37 SMEs in round 1 to 25 in round 3, reflecting a common issue in Delphi studies as participants become fatigued or disengaged over time.

Additionally, while the Delphi method is advantageous for fostering agreement among diverse experts, it carries the assumption that an SME can be universally knowledgeable across all cancer types. Most SMEs possess specialised knowledge in specific cancers, leading to a potential knowledge gap in understanding and assessing genes outside their primary focus. Although the Delphi process encourages cross-disciplinary input, it remains challenging to ensure that all contributors possess a comprehensive understanding of genes relevant across cancers, particularly for adult versus paediatric tumour types. This variability in expertise could impact the robustness of gene evaluations, as some genes may receive disproportionate consideration based on individual SME’s backgrounds. This underrepresentation of certain expertise likely explains why a subset of genes related to paediatric and brain cancers that are uses in clinical trial selection have not been included within this panel.

A key limitation of this panel is that its small size inherently restricts accurate estimation of tumour mutation burden (TMB). While large, clinically validated panels such as FoundationOne CDx (324 genes) or MSK-IMPACT (468 genes) have been shown to provide reliable TMB estimates that correlate with whole-exome sequencing, smaller panels like this proposed 99-gene panel may underestimate TMB or fail to capture its full variability [7, 8]. As a result, caution is warranted when using this panel to guide therapies where TMB is a key biomarker. In clinical settings where TMB is critical for treatment decision-making, supplemental assays may be required to ensure accurate assessment, while the 99-gene panel can still serve as an efficient tool to triage tumours for research-oriented clinical trials [9–18].

A final limitation is the omission of potentially clinically important genomic signatures, such as homologous recombination deficiency (HRD). Whilst TMB and MSI were both considered for this panel, HRD was not. It is critical to acknowledge the clinical utility of HRD in ovarian, breast, pancreatic and prostate cancers. However, its omission from the recommendation within this paper does not definitively deny its position on a future panel. This reflects an area of improvement for the proposed panel.

This research supports the case for a unified pan-cancer panel within the NHS, laying the groundwork for the genes and mutation types to screen for in a standardised pan-cancer gene panel that could be used consistently across NHS genomics laboratories. By establishing a shared framework for genetic testing, this study offers a blueprint for creating a more cohesive approach to cancer diagnostics and personalised care within the NHS, cancer research and beyond.

## Conclusion

This study successfully developed a consensus-driven, ECMC-approved pan-cancer gene panel through a structured Delphi process involving academic and industry experts across the UK. The final panel of 99 genes, along with TMB, MSI, and selected SVs, CNVs, and fusions, offers a high-utility, standardised framework for broad-spectrum genomic screening. By aligning expert opinion across tumour types, this panel supports equitable patient access, enhances research trial stratification, and paves the way for a unified approach to precision oncology within the NHS.

## Supplementary information

Supplementary table 1

## Data Availability

The datasets generated and/or analysed during the current study comprise aggregate Delphi survey responses from academic experts. These data are not publicly available due to the confidential nature of expert responses and the terms of participant consent, but anonymised data are available from the corresponding author on reasonable request.
