# Supplementary material for: Experimental Cancer Medicine Centre (ECMC) network proposal for a consensus gene panel for pan-cancer sequencing: a Delphi methodology
Source: Br J Cancer. 2025 Nov 8;134(2):289–94. doi: 10.1038/s41416-025-03252-6 (PMC12820137; doi:10.1038/s41416-025-03252-6)
Supplement: Supplementary file 1 — Supplementary table 1 [file 41416_2025_3252_MOESM1_ESM.docx]

| **Gene** | **Predictive of response** | **Prognostic** | **Cancer predisposition risks** | **Particular tumours of relevance** |
| --- | --- | --- | --- | --- |
| *AKT1* | Guides use of *AKT* inhibitors | Poor prognostic marker in hormone receptor positive / HER2-positive breast cancer |  | Breast |
| *AKT2* |  | *AKT2* amplification has been associated with aggressive phenotypes in breast, ovarian and PDAC |  | Breast, ovarian and PDAC |
| *AKT3* |  | *AKT3* alterations are linked to melanoma progression and resistance to certain treatments |  | Melanoma |
| *ALK* | Guides use of *ALK* inhibitors | Presence of *ALK* fusion is associated with improved prognosis |  | NSCLC |
| *APC* |  | Associated with poor prognosis, particularly with worse immunotherapy outcomes as correlates with a lower TMB | Identify germline variants in *APC* to diagnose familial adenomatous polyposis (FAP), which carries a high risk for early-onset colorectal cancer | CRC |
| *AR* |  | Androgen receptor (*AR*) mutations offer a poor prognostic survival outcomes due to resistance to anti-hormonal therapies |  | Prostate |
| *ARID1A* |  | Generally associated with poor prognosis for overall survival, however there are some tumour-specific exceptions |  | Tumour agnostic (solid tumours) |
| *ARID1B* |  | Can be associated with improved prognosis (e.g. progression-free survival and relapse-free survival), however there are some tumour-specific exceptions |  | Tumour agnostic (solid tumours) |
| *ATM* | Supports consideration of DNA damage repair targeting agents, such as ATR inhibitors or WEE1 inhibitors | Poor prognosis in breast cancer | Identify germline variants in *ATM*, which carries a high risk for breast, ovarian, prostate and pancreatic. | Tumour agnostic (solid tumours) |
| *ATR* | Supports consideration of DNA damage repair targeting agents, such as ATM inhibitors or WEE1 or PARP inhibitors |  |  | Broad range of solid tumours, particularly ovarian |
| *ATRX* | Supports consideration of DNA damage repair targeting agents, such as WEE1 or PARP inhibitors | Positive prognostic indicator of overall survival in sarcoma and low-grade glioma. Associated with inferior outcome in neuroblastoma. |  | Neuroblastoma, pancreatic NETs, osteosarcomas and gliomas. |
| *BAP1* |  | *BAP1* carries poor survival outcomes in uveal melanoma and RCC but improved outcomes in mesothelioma | *BAP1* tumour predisposition syndrome | Uveal melanoma, malignant pleural and peritoneal mesothelioma and RCC. |
| *BCL2* |  | Associated with poor survival outcomes in follicular lymphoma. *BCL2* mutations may cause resistance to BCL2 inhibitors in CLL/AML. |  | Follicular lymphoma, AML, CLL |
| *BRAF* | Guides use of V600E *BRAF* inhibitors | Associated with poor prognosis in CRC and melanoma (despite targeted treatments for the latter tumour type) |  | NSCLC, melanoma, paediatric low-grade glioma, CRC |
| *BRCA1* | Supports use of PARP inhibitors | Poorer prognosis in triple negative breast cancer | Identify germline variants in *BRCA1*, which carries a high risk for breast, ovarian and prostate cancer | Breast, ovarian, prostate |
| *BRCA2* | Supports use of PARP inhibitors.  Some reports of increased immune checkpoint blockade sensitivity in combination with TMB |  | Identify germline variants in *BRCA2*, which carries a high risk for breast, ovarian, prostate and pancreatic cancer | Breast, ovarian, prostate |
| *BRIP1* | Suggests use of DNA damage repair targeting agents |  | Identify germline variants in *BRIP1*, which carries a high risk for ovarian cancer | Prostate, breast, ovarian |
| *CCND1* |  | High expression of CCND1 was associated with a poor prognosis in HNSCC and PDAC, while a high expression of *CCND1* was associated with a good prognosis in clear cell RCC, stomach adenocarcinoma and endometrial carcinoma. |  | Tumour agnostic (solid tumours) |
| *CCND2* |  | Lower *CCND2* expression correlates with shorter overall survival |  | NSCLC |
| *CCNE1* |  | *CCNE1 amplification or overexpression is linked to poor overall survival in triple negative breast cancer* |  | Breast |
| *CDH1* |  |  | Identify germline variants in *CDH1*, which carries a high risk for hereditary diffuse gastric cancer and lobular breast cancer | Breast, stomach primarily, however also seen in CRC, thyroid, ovarian, endometrial and prostate |
| *CDK4* |  | Amplification may suggest aggressiveness |  | Breast, neuroblastoma |
| *CDK6* |  | Amplification may suggest aggressiveness |  | Breast |
| *CDK12* | Supports consideration of PARP inhibitors in prostate cancer | Associated with poor prognosis in low-grade glioma, melanoma, mesothelioma and PDAC |  | Tumour agnostic (solid tumours) |
| *CDKN2A* |  | Negative prognostic overall survival indicator in PDAC | Identify germline variants in *CDKN2A*, which carries a high risk for melanoma and PDAC | Melanoma, PDAC, glioma and ALL |
| *CDKN2B* |  | Deletions or silencing of *CDKN2B* are associated with poor prognosis in multiple cancers, including glioblastoma, ALL (adult and child), and PDAC |  | Glioblastoma, ALL and PDAC |
| *CHEK1* | Supports consideration of DNA Damage Repair targeting agents e.g. ATM inhibitors or PARP inhibitors in prostate cancer | Poor prognosis reported in breast, oesophageal SCC, gastric and CRC |  | Tumour agnostic (solid tumours) |
| *CHEK2* | Supports consideration of DNA Damage Repair targeting agents e.g. ATM inhibitors or PARP inhibitors in prostate cancer | Poor prognosis reported in symptomatic early-onset breast cancer | Identify germline variants in *CHEK2*, which carries a high risk for breast and prostate cancer. | Breast, prostate |
| *CTNNB1* |  | Favourable prognosis in HCC, poor prognosis in endometrial cancer and desmoid tumours |  | HCC, endometrial cancer, desmoid tumours, adrenocortical carcinoma |
| *EGFR* | Guides use of *EGFR* tyrosine kinase inhibitors |  |  | NSCLC, glioblastoma, HNSCC |
| *ERBB2* | Guides of use of anti-HER2 therapy | Presence of *ERRB2* amplification in gastric and breast cancers implies aggressive cancers however offers targeted therapies which improve prognosis compared to HER2 negative cancers |  | Breast, CRC and gastric cancer |
| *ERBB3* | May guide use of anti-HER3 therapies in the future | *ERBB3* overexpression often correlates with poor prognosis in breast, ovarian, gastric, and CRC. |  | Breast, ovarian, gastric and CRC |
| *ERBB4* |  | Elevated ERBB4 expression offers a more favourable prognosis in urothelial cancers and intrahepatic CCAs; however offers poorer prognosis in gastric cancer |  | Urothelial cancer, CCA, gastric cancer |
| *ESR1* | Guides the choice of second-line endocrine therapy in metastatic hormone receptor positive breast cancer | *ESR1* mutation associated with poor prognosis in breast cancer due to resistance to endocrine therapy |  | Breast |
| *EZH2* | Guides use of *EZH2* inhibitors in relapsed follicular lymphoma and epithelioid sarcoma | Overexpression in solid tumours carries an unfavourable prognosis |  | Follicular lymphoma, DLBCL, AML and MDS Tumour agnostic in solid tumours. |
| *FANCA* | May support consideration of DNA repair targeting agents (e.g. PARP inhibitors and platinum-based chemotherapy) |  | Identify germline variants in *FANCA*, which carries a high risk for breast and ovarian cancer (Fanconi anaemia) | AML and MDS, prostate, breast, ovarian and PDAC |
| *FANCC* | May support consideration of DNA repair targeting agents (e.g. PARP inhibitors and platinum-based chemotherapy) |  | Identify germline variants in *FANCA*, which carries a high risk for breast and ovarian cancer (Fanconi anaemia) | AML and MDS, prostate, breast, ovarian and PDAC |
| *FGFR1* |  | *FGFR1* amplification correlates with more aggressive disease and worse prognosis |  | NSCLC, breast, glioblastoma, bladder, multiple myeloma, CCA, gastric cancer |
| *FGFR2* | Guides use of *FGFR2* fusion inhibitors, particularly in cholangiocarcinoma | Associated with worse prognosis in breast, gastric and endometrial cancer |  | CCA |
| *FGFR3* | Guides use of *FGFR3* mutations or fusions |  |  | Tumour agnostic (solid tumours) but approved in urothelial carcinoma |
| *FGFR4* | May guide use of selective FGFR4 inhibitors | Poor survival outcomes in HCC and breast cancer |  | HCC, breast cancer, rhabdomyosarcoma, CRC, NSCLC and gastric cancer |
| *FLT1* |  | High *FLT1* expression may correlate with worse prognosis or more aggressive disease in breast, colorectal, gastric, and pancreatic cancers. |  | Tumour agnostic (solid tumours) |
| *FLT3* | Guides use of *FLT3* inhibitors in *FLT3*-mutated AML | Mutations in key domains of *FLT3* carry high relapse risk and worse overall survival |  | AML and MDS |
| *HLAA* |  | High *HLAA* expression is associated with a hot tumour microenvironment and good prognosis |  | Tumour agnostic (solid tumours) |
| *HRAS* | May guide use of *HRAS* inhibitors for *HRAS*-mutated head and neck squamous cell carcinoma |  |  | Urothelial cancer, HNSCC, follicular thyroid cancer and melanoma |
| *IDH1* | Guides use of *IDH1* inhibitors for relapse/refractory AML and CCA | Mutations may suggest a favourable prognosis in gliomas and AML |  | Diffuse gliomas, AML CCA |
| *IDH2* | Guides use of *IDH2* inhibitors for relapse/refractory *IDH-2* mutant AML |  |  | AML, cholangiocarcinoma |
| *JAK1* | Guides use of JAK inhibitor in high-risk myelofibrosis with activating mutations in *JAK1* | Activating *JAK1* mutations are associated with high-risk disease; loss of function mutations in *JAK1* are associated with primary resistance to PD-1 blockage |  | T-cell ALL, myeloproliferative neoplasms, urothelial cancer, endometrial cancer, melanoma |
| *JAK2* | Guides use of JAK inhibitor in high-risk myelofibrosis | Loss of function mutations in *JAK2* are associated with primary resistance to PD-1 blockage |  | Melanoma, myelofibrosis |
| *JAK3* |  | Activating *JAK3* mutations may correlate with high-risk disease |  | T-ALL, T cell lymphoma, AML |
| *KIT* | Guides use of *KIT* inhibitors for GI stromal tumours and melanoma) | Exon 11 mutations carry a better response to imatinib, whereas exon 9 carries a higher risk of progression |  | GISTs, melanoma, AML |
| *KRAS* | Guides use of *EGFR* inhibitors if *KRAS* is absent of mutations (wild-type) in CRC.  Guides use of KRAS G12C/G12D and panKRAS inhibitors. | Associated with more aggressive disease in PDAC and CRC |  | Colorectal, NSCLC, PDAC |
| *MEN1* |  |  | Identify germline variants in *MEN1*, which carries risk for *MEN1*-associated tumours (e.g. gastrinomas, insulinomas and VIPomas) | Endocrine tumours |
| *MET* | Guides use of *MET* inhibitors | *MET* amplification is associated with poor prognosis in gastric cancer and NSCLC |  | NSCLC, melanoma, gastric cancer |
| *MLH1* | Consider immune checkpoint blockade or WRN1 inhibition in MSI high tumours | Generally, carries a better prognosis than microsatellite stable tumours in CRC setting | Identify germline variants in *MLH1* which is diagnostic of Lynch syndrome (mismatch repair deficiency / microsatellite instability) | CRC, endometrial, gastric, ovarian and urothelial cancers |
| *MSH2* | Consider immune checkpoint blockade or WRN1 inhibition in MSI high tumours |  | Identify germline variants in *MSH2* which is strongly suggestive of Lynch syndrome (mismatch repair deficiency / microsatellite instability) | CRC, endometrial, gastric, ovarian and urothelial cancers |
| *MSH3* |  |  | Not considered a primary diagnostic marker of Lynch syndrome | CRC, endometrial, gastric, ovarian and urothelial cancers |
| *MSH6* |  |  | Identify germline variants in *MSH6*, which causes Lynch syndrome (mismatch repair deficiency / microsatellite instability) | Later-onset endometrial cancer, CRC, urothelial, gastric, ovarian |
| *MTOR* |  | *MTOR* pathway activation may be associated with aggressive disease and poor prognosis |  | Clear cell RCC, tuberous-sclerosis associated RCC |
| *MUTYH* |  |  | Germline biallelic or monoallelic loss-of-function mutations associated with colorectal cancer and duodenal adenomas | CRC |
| *MYC* |  | *MYC* amplification/rearrangement often indicates aggressive disease with poor prognosis | *MYC* rearrangement is part of lymphoma classification | Burkitt lymphoma, DLBCL, ALL, breast, neuroblastoma, SCLC |
| *MYCN* |  | MYCN amplification associated with high-risk, aggressive disease in neuroblastoma | *MYCN* testing may be part of neuroblastoma diagnosis | Neuroblastoma, medulloblastoma, SCLC, rhabdomyosarcoma |
| *NF1* |  |  | Primarily used in germline NF1 syndromes | Glioblastoma, low-grade glioma, melanoma, lung and breast cancer |
| *NF2* |  |  | Primarily used in germline NF2 syndromes | Mesothelioma, meningioma, schwannoma |
| *NRAS* | Guides use of *EGFR* inhibitors if *NRAS* is absent of mutations (wild-type) in CRC  Guides use of *MEK* inhibitors in melanoma | *NRAS-*mutant melanoma may be associated with aggressive disease and poorer prognosis |  | Melanoma, AML, T-ALL, CRC |
| *NRG1* | *NRG1* fusions may activate *ERBB2/ERBB3* signalling offering therapeutic opportunities |  |  | Lung |
| *NTRK1* | Guides use of *NTRK* fusion inhibitors |  |  | Tumour agnostic (solid tumours) |
| *NTRK2* | Guides use of *NTRK* fusion inhibitors |  |  | Tumour agnostic (solid tumours) |
| *NTRK3* | Guides use of *NTRK* fusion inhibitors |  |  | Tumour agnostic (solid tumours) |
| *PALB2* | Guides use of PARP inhibitors and platinum-based chemotherapy given the inherent homologous recombination deficiency (HRD) associated with *PALB2* mutations |  | Germline *PALB2* mutations confer increase risk of pancreatic and ovarian cancer | Breast, prostate, PDAC and ovarian |
| *PARP1* |  | May predict resistance to PARP inhibitors |  | Breast, ovarian, prostate and PDAC |
| *PDGFRA* | Guides use of imatinib in most PDGFRA-mutant GISTs (other than D842V mutation) and avapritinib in D842V mutant GISTs |  |  | GISTs, gliomas |
| *PDGFRB* | Guides use of imatinib in PDGFRB fusion-positive neoplasms |  |  | Myeloid/lymphoid neoplasms with eosinophilia |
| *PIK3CA* | Guides use of *PIK3CA* inhibitors in breast cancer | Carries a poor prognosis in breast cancer |  | Breast, CRC, endometrial, cervical and HNSCC |
| *PMS1* | Consider immune checkpoint blockade or WRN1 inhibition in MSI high tumours |  | Contribute to a Lynch-like syndrome | Lynch-like CRC, endometrial, gastric, ovarian and urothelial cancers |
| *PMS2* | Consider immune checkpoint blockade or WRN1 inhibition in MSI high tumours |  | Identify germline variants in *PMS2*, which causes a mild phenotype of Lynch syndrome (mismatch repair deficiency / microsatellite instability) | CRC, endometrial, ovarian, gastric, urothelial cancers |
| *POLE* | Consider immune checkpoint blockade | *POLE*-mutant tumours carry an excellent prognosis as they are associated with high tumour mutational burden and strong anti-tumour immune responses |  | CRC, endometrial, glioblastoma |
| *PTEN* |  | *PTEN* carries poor prognosis in glioblastoma, breast and prostate | Identify germline variants in *PTEN*, which causes *PTEN* hamartoma tumour syndrome (PHTS), e.g. Cowden syndrome | Breast, endometrial, prostate, glioblastoma |
| *RAD51* | Low *RAD51* function indicates homologous recombination deficiency and indicates a sensitivity to PARP inhibitors | Overexpression carries poor prognosis in breast and ovarian cancer |  | Breast, ovarian, PDAC |
| *RAD51B* | Supports consideration of DNA damage repair targeting agents |  |  | Breast, ovarian, prostate |
| *RAD51C* | Supports consideration of DNA damage repair targeting agents |  |  | Ovarian, breast, prostate |
| *RAD51D* | Supports consideration of DNA damage repair targeting agents |  |  | Ovarian, breast, prostate |
| *RB1* |  | *RB1* loss in small cell lung cancer indicates aggressive disease and poor prognosis | Identify germline variants in *RB1*, which causes hereditary retinoblastoma | Retinoblastoma, SCLC, osteosarcoma, breast, prostate and urothelial cancer |
| *RET* | Guides use of *RET* fusion inhibitors |  |  | Medullary thyroid carcinoma, papillary thyroid carcinoma, NSCLC |
| *ROS1* | Guides use of *ROS1* inhibitors |  |  | NSCLC, CCA, glioblastoma |
| *SETD2* |  | *SETD2* carries a poor prognosis in RCC and glioblastoma |  | RCC, AML, paediatric high-grade gliomas |
| *SMAD4* |  | Somatic loss of *SMAD4* is associated with chemoresistance in PDAC and CRC | Identify germline variants in *SMAD4*, which causes juvenile polyposis syndrome (JPS) | PDAC, CRC |
| *SMARCA4* |  | *SMARCA4* carries a very poor prognosis with an aggressive course | IHC loss of BRG1 protein is diagnostic for *SMARCA4-deficient tumours* | Undifferentiated thoracic tumours, small cell carcinoma of the ovary, hypercalcaemia type (SCCOHT), NSCLC |
| *SMARCB1* |  | *SMARCB1* deficiency is associated with highly aggressive tumours and poor prognosis | IHC loss of INI1 protein is diagnostic for *SMARCB1-deficient tumours* | Rhabdoid tumours, epithelioid sarcoma |
| *STK11* |  | *STK11* mutations are associated with more aggressive tumours and a worse prognosis, associated with resistance to immune checkpoint therapy | Identify germline variants in *STK11*, which causes Peutz-Jeghers syndrome (PJS) | NSCLC, cervical, PDAC and CRC |
| *TERT* |  | *TERT* promoter mutations indicate aggressive tumour behaviour and poor prognosis | *TERT* mutation status helps subtype classification in gliomas | Gliomas, melanoma, papillary and anaplastic thyroid cancer, urothelial |
| *TET2* |  |  | Aids diagnosis for AML, MDS and CMML | AML, MDS, CMML |
| *TP53* | Certain variants under investigation using TP53 targeting agents | Generally, indicates a poor prognosis with aggressive disease | Identify germline variants in *TP53*, which causes Li-Fraumeni syndrome | Tumour agnostic (solid tumours) |
| *TSC1* | May guide use of mTOR inhibitors |  | Identify germline variants in *TSC1*, which causes tuberous sclerosis complex (TSC) | Urothelial and RCC |
| *TSC2* | May guide use of mTOR inhibitors |  | Identify germline variants in *TSC2*, which causes tuberous sclerosis complex (TSC) | RCC, perivascular epithelioid cell tumours |
| *VEGFA* | May guide use of anti-angiogenic therapy | High *VEGFA* expression is often associated with aggressive disease and poor prognosis |  | Tumour agnostic (solid tumours) |
| *VHL* | May guide use of anti-angiogenic therapy |  | Identify germline variants in *VHL*, which causes Von-Hippel-Lindau syndrome | Clear cell RCC |

**Supplementary table 1:** Clinical applications of mutations within genes included in the ECMC 99-gene panel. Acute myeloid leukaemia (AML), cholangiocarcinoma (CCA), chronic lymphocytic leukaemia (CLL), chronic myeloid leukaemia (CML), chronic myelomonocytic leukaemia (CMML), colorectal carcinoma (CRC), diffuse large B-cell lymphoma (DLBCL), gastrointestinal stromal tumours (GISTs), head and neck small cell carcinoma (HNSCC), myelodysplastic syndrome (MDS), neuroendocrine tumour (NET), non-small cell lung carcinoma (NSCLC), pancreatic ductal adenocarcinoma (PDAC), renal cell carcinoma (RCC), small cell lung carcinoma (SCLC),squamous cell carcinoma (SCC) [8][9][10][11][12][13][14][15][16][17][18].
